# Higher Education Management and Student Achievement Assessment Method Based on Clustering Algorithm

**DOI:** 10.1155/2022/4703975

**Published:** 2022-07-04

**Authors:** Zhihui Wang

**Affiliations:** Huanghe S&T University, Zhengzhou 450005, China

## Abstract

Monitoring and guiding instructional management require student performance evaluation. Traditional evaluation and analysis methods based on absolute scores, on the other hand, have certain flaws and are unable to fully reflect the information contained in student performance, thus limiting the impact of student performance evaluation on teaching and learning management. Data mining is regarded as the backbone technology for future information processing, and it introduces a new concept to the way humans use data. Schools must analyse and evaluate the performance of students in the same grade level and secondary school in a timely and staged manner. Clustering is a type of data mining that uses similarity rules to classify sample data into groups with a high degree of similarity. To address the difficulties caused by the wide variation in course difficulty in student performance evaluation, a method based on the *K*-means clustering algorithm is proposed. The *K*-means algorithm and the improved K-means algorithm with student information are investigated. The test results showed that the *K*-means clustering algorithm, the improved algorithm in this paper, and the fast global mean clustering algorithm all cluster the same randomly generated data set with noisy points, but the clustering time of the algorithm in this paper is only 0.04, which has obvious advantages. As a result, the clustering algorithm-based higher education management and student performance evaluation mechanism provides some insights for future research on student learning patterns. It is hoped that instructional administrators will gain a better understanding of students' learning characteristics so that they can better guide their teaching.

## 1. Introduction

Humans have greatly improved their ability to collect, process, organise, and produce information using information technology as the world moves toward an information society [1]. This has resulted in the creation of tens of thousands of databases of various types, all of which play an important role in scientific research, technology development, production management, market expansion, business operations, government offices, and other areas [2, 3]. Schools now have a variety of systems and databases that collect a large amount of data on the student achievement. Staff can only obtain a small amount of information through simple statistics of Excel tools due to a lack of relevant mining knowledge and technology, and the information hidden in these large amounts of data cannot be applied [4]. The globalised information reserve is expanding, and the amount of information available to people is expanding as well [5]. However, people's thirst for knowledge grows as they learn how to quickly find the information they need from such a vast amount of data [6].

However, there are some issues with evaluating teaching quality in universities in general. Traditional teaching quality evaluation methods have a number of flaws, and the results obtained are not scientific or accurate enough [7]. Various systems and databases are used by universities to collect large amounts of data [8]. Through a series of scientific and systematic evaluations, the primary goal of evaluation is to encourage universities to optimise their own resources, improve efficiency, improve academic research and talent training quality, and realise the social functions of universities [9]. Higher education management is a critical aspect of higher education operations, as it is linked to the quality and efficiency of the institution [10]. Data structure can be classified without prior classification using the cluster analysis method, revealing the inherent differences and connections of objective things [11]. Massive data analysis can provide useful guidelines for business management, project development, Internet finance, and efficient scientific research, allowing data resources to be fully utilised [12].

There are several clustering methods available, the most basic of which is partitioned clustering [13, 14]. Partitioned clustering tries to divide a data set into disjoint subsets in order to find the best clustering criteria. Researchers have divided clustering analysis into four categories to address these specific practical issues: partition-based methods, hierarchical methods, density-based methods, and grid-based methods [15]. Clustering algorithms are an important step in the modernization of education, which promotes the cultivation of innovative talents and improves the quality of all people. It completes the digital network automation of educational teaching management, campus life management, and other activities, encourages the growth of the educational information industry and the effective use of educational big data, and gradually develops an innovative educational management model that adapts to the needs of social informatization. Among them, the *K*-means algorithm is a traditional clustering algorithm based on division that performs clustering through continuous iteration. When the algorithm reaches the end condition, the iterative process is completed and the clustering results are output. The following are the paper's unique features. (1) In this study, data mining technology [16] is used to analyse students' academic performance data using fuzzy clustering, and “teaching” and “learning” are organically combined. (2) We can identify a number of undesirable phenomena in teaching activities, judge whether the teaching methods are reasonable, consider whether existing teaching theories can support classroom teaching, and assist in the development of teaching theories by evaluating classroom teaching quality. (3) Analyzing higher education management and student performance evaluation mechanisms using clustering algorithms. Data mining techniques can be used to describe and classify data, predict future behaviors and trends, and solve the time-consuming problem of traditional data analysis.

## 2. Related Work

### 2.1. Higher Education Management and Student Achievement Evaluation

Exams and teaching are inextricably linked in school education, and exam results are crucial in determining how well the students learn. Colleges and universities frequently use the average or total score as the grade standard when evaluating the general qualifications of students. This evaluation method is simple and easy to use in real-world teaching feedback, but it ignores the singularity and one-sidedness of the evaluation results caused by the test paper difficulty. As a result, we should investigate the current state of academic achievement evaluation in China's secondary schools, use data mining technology to investigate models and methods suitable for secondary school academic achievement analysis and comprehensive evaluation, and infuse new thinking into secondary school education quality comprehensive evaluation reform.

Omar's academic achievement evaluation should pay special attention to the process evaluation, which is a dynamic evaluation. It should not only be the evaluation of learning achievement, but should include learning attitude, behavior, and habit in the evaluation system. Li et al. judged from three aspects of evaluation view, evaluation method, and evaluation process, and proposed the evaluation model of two-way evaluation subject, comprehensive evaluation method, and multi-way evaluation perspective [17]. Goh et al.‘s school uses test scores to evaluate students in a single way, which causes valuable data resources of wasting. It is recommended to use better models and software to analyse the scores [18]. Omolewa et al. analysed evaluation activities in terms of pedagogical elements and constructed a new evaluation model in terms of objectives, form, and content [19]. Wiechetek and Pastuszak argue that the evaluation of students' academic performance should tend to be diversified, focusing not only on their creativity, but also on their behavioral habits and values [20].

Through the comprehensive evaluation of students' academic performance, we can identify the gaps between students and classes, and then recognize the differences between the level of discipline teachers' teaching and classroom management of classroom teachers, so as to guide the next stage of discipline teaching and classroom management more effectively. In addition, through the analysis and comparison of various types of students' performance, corresponding improvement suggestions are given, which provide a theoretical basis for students' performance evaluation, personalized development and teachers' differentiated teaching.

### 2.2. Clustering Algorithm

Evaluation is an important tool for assessing and monitoring educational quality, as well as guiding teachers' teaching practices and encouraging students to pursue learning. Traditional performance analysis methods, on the other hand, are unable to objectively and accurately reflect the relative distribution of students' performance and the classification of their learning situation. It can only perform statistical analysis on the scores and knowledge points on candidates' test papers, and it cannot mine and analyse large amounts of data from the remote examination system well. If we are to make full use of the vast amount of data accumulated in the past and dig them out, we must first understand the characteristics of students' learning. We can analyse these academic performance data in depth as school administrators, classroom teachers, and subject teachers, discarding the “criticism” brought on by traditional performance ranking and exploring the data's value in providing powerful support for the implementation of the education strategy of “teaching according to students' abilities”

Davenport constructed a variable precision rough set model based on relational algorithms and performed a preliminary analysis of student performance [21]. Anoopkumar and Rahman et al. proposed a nonparametric clustering method for categorical attribute data, which became the *K*-modes-CGC algorithm, similar to the traditional *K*-means algorithm for numerical data [22]. Mayureshwar et al. used data mining techniques for distance learning in educational systems. In a distance personalized learning system, resources can be allocated for the individual learning needs of the students [23]. The Weka tool built by Belan Velasco et al. integrates various data mining algorithms into an interactive interface, which includes a variety of data preprocessing, categorization, degradation, regression, clustering, correlation rules, and optimization [24].

The process of teaching informatization has produced a vast volume of educational data, and these valid records are a reliable support for us to carry out the next stage of data mining. Clustering to several classes or clusters of data objects enables high similarity among objects in the identical clusters, but with significant differences among objects in various clusters. Through aggregation, one can identify dense and sparse regions, and discover global distribution patterns and interesting relationships among data attributes.

## 3. Thoughts on Higher Education Management and Student Achievement Evaluation Based on Clustering Algorithm

### 3.1. Performance Evaluation Scheme Based on Clustering Algorithm

Student performance evaluation is a kind of educational evaluation, a key link and important element of teaching and instructional management, and the most important source of information for teaching quality evaluation. Clustering analysis, as an exploratory analysis method, is widely used in pattern recognition [25, 26], computer vision [27, 28], data mining, and other fields. Its purpose is to divide a physical or abstract set of objects into several subsets based on the principle of similarity, and analyse the intrinsic connections, patterns, and characteristics of data objects within each subset. The sum of data corresponding to each clustering element is calculated by dividing the data of each element by the sum of data of this element, i.e.,

(1) $$\begin{array}{c} x_{ij}^{\prime }=\frac{x_{ij}}{\sum_{i=1}^{m}x_{ij}},\left(i=1,2,\ldots ,m;j=1,2,\ldots ,n\right). \end{array}$$

**Figure 1:**
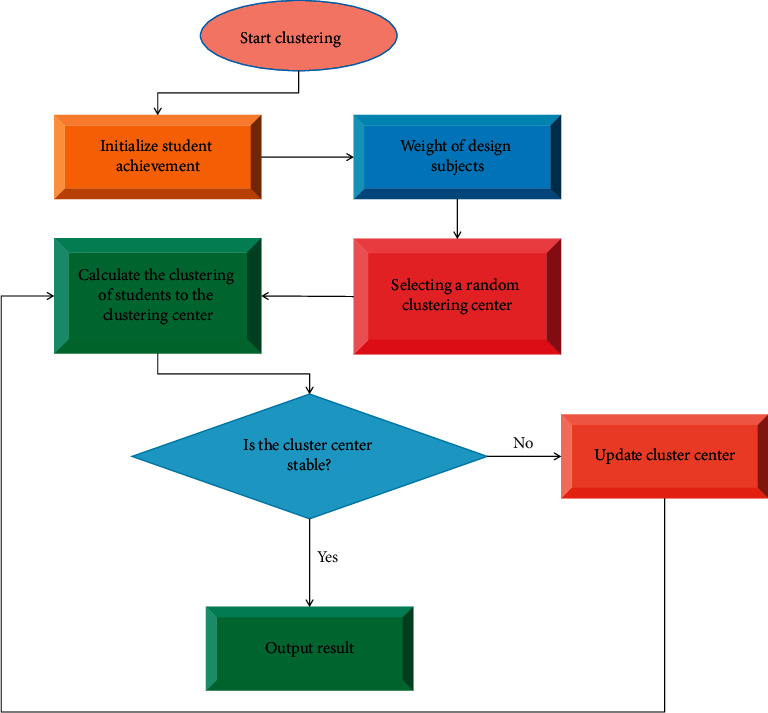
*K*-means algorithm studies the process of students' achievement.

Scientific evaluation of students' learning performance can help teachers and parents to grasp students' learning, and teachers and parents can make corresponding adjustments to the teaching and supervision of students' learning based on the evaluation results. The flowchart of studying student performance with *K*-means algorithm is shown in Figure 1.

The first step is data collection. In order to ensure the completeness and accuracy of the data, the original data sources must be well selected and organised. The original data source is input and with the participation of experts, it is decided which attribute features to use to describe the nature and structure of the data source. Feature extraction outputs a matrix where each row represents a sample and each column represents a feature index variable. The new data obtained by this normalization has a maximum value of 1 for each element and all other values are less than 2. Therefore, the normalization is poor, i.e.,

(2) $$\begin{array}{c} x_{ij}=\frac{x_{ij}-\text{min}\left(x_{ij}\right)}{\max\left(x_{ij}\right)-\min\left(x_{ij}\right)},\left(i=1,2,\ldots ,m;j=1,2,\ldots ,n\right). \end{array}$$

**Figure 2:**
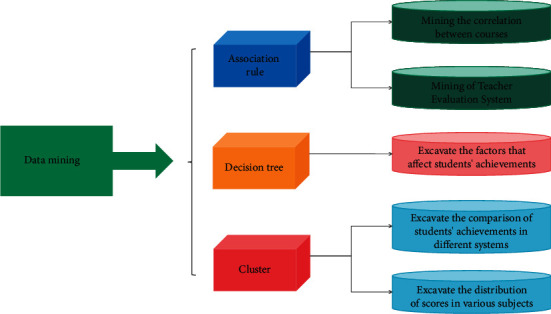
Data mining process.

The representative data among them are extracted, and these extracted data are what are normally called sample data in data-related work [29]. Each data object is considered as a point in the d dimensional space, and then a certain distance is used to represent the similarity between the data objects. Data objects that are closer are similar in nature, while data objects that are farther away are very different. This is a phase of great workload and time. The data mining process is shown in Figure 2.

Next is data preprocessing, through the four steps of data review, data cleaning, data conversion, and data verification, the data is preprocessed to resolve data conflicts and data inconsistencies, and finally form student reports. The most commonly used clustering standard function, the error sum of squares, is applicable to all sample distributions with dense samples and small differences in sample size:

(3) $$\begin{array}{c} J_{c}={\sum_{j=1}^{c}\sum_{k=1}^{n_{j}}\left\|x_{k}-m_{j}\right\|}_{2}. \end{array}$$

The quality of data mining results will be adversely affected if the data input to the database has anomalous data, irrelevant fields, or even conflicting fields, improper data coding methods, and other anomalies [30, 31]. The sample matrix extracted in the first step is input to the clustering, and the individual sample is imagined as a point in the feature variable. The mean squared deviation is generally used as the standard measure function and is defined as follows:

(4) $$\begin{array}{c} E={\sum_{i=1}^{k}\sum_{peC_{i}}\left|p-m_{i}\right|}_{2}. \end{array}$$

Since there are many indexes in the raw data, each index may have different dimensions and orders of magnitude. If the calculation is done directly using the raw data, it is likely that a large number of indexes will directly affect the clustering results. By definition, those data that are not suitable for training and learning are extracted from the sample data and excluded from the system. It should be noted that these data that need to be cleaned up generally refer to incomplete data. To accommodate the introduction of fuzzy partitioning [32, 33], the affiliation matrix allows the elements to have values between 0 and 1. However, using the normalization rules, the sum of the affiliations of the data set is always equal to 1:

(5) $$\begin{array}{c} \sum_{i=1}^{c}u_{ij}=1,\forall j=1,\ldots ,n. \end{array}$$

Finally, after performing the clustering algorithm to determine the mining task, the K-means clustering algorithm is written in matlab to implement the processing of the student achievement analysis. If the form of data used for mining does not match the characteristics of mining and they are necessary, in the face of this situation, what needs to be done is data conversion, conversion of display formats or coding formats, etc. After obtaining the clustering spectrum map in the second step, the expert can select the appropriate threshold value according to the particular applied situation. The data requires “sorting” and “filtering” to prevent anomalies in the input data, which can enhance the effectiveness of data mining and the accuracy of mining results. When two vectors are similar in direction, the cosine of the angle is larger, and vice versa, it is smaller. When two vectors are parallel, the cosine of the angle is 1, and when the vectors are orthogonal, the cosine of the angle is 0.

### 3.2. Higher Education Management Method Based on Clustering Algorithm

**Figure 3:**
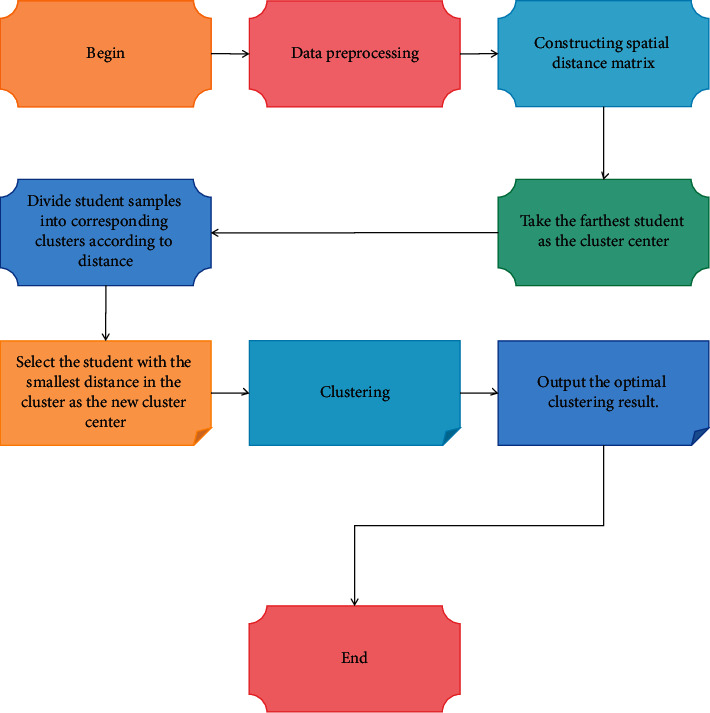
Clustering process of higher education management.

As far as the school is concerned, the school needs data mining technology so that it can see the differences with other universities and enhance its competitiveness. It also needs a detailed and comprehensive understanding of its students' characteristics to improve their academic performance and practical skills, which in turn improves its teaching quality. The whole clustering higher education management process is divided into three parts: data preprocessing, multiple clustering result comparison, and optimal clustering result output, as shown in Figure 3.

First, you can tap into students' learning patterns, divide them into different groups, designate reasonable learning plans based on guidance, understand the characteristics of students in the groups, and strive for effective learning strategies. In the evaluation of student achievement, each class is an achievement group. Different classes divide each achievement group accordingly, corresponding to the central scores given to the different achievement groups. These center scores are one of the reference criteria for the classification of student achievement. We call the distance between two class centers the inter-class distance. If *C*_*i*_ is the clustered sample, define the class centers of *C*_*i*_ as follows:

(6) $$\begin{array}{c} \bar{x_{i}}=\frac{1}{n_{i}}\sum_{x\subset C_{i}}x, \end{array}$$

*n*_*i*_ is the number of points in the *i*th cluster.

In practical problem analysis, different fuzzy similarity matrix construction methods may lead to different clustering results. It is necessary to decide the best fuzzy similarity matrix construction method based on the comparison results of the nature of the problem.

Next are class management, developing lesson plans, prescheduling, group class selection, managing scheduling, managing student attendance, and open control of teaching services. The total objective of evaluating teachers' teaching is divided into different secondary objectives, such as teaching attitude and teaching ability. Each secondary objective is then decomposed into several influencing factors, thus forming a hierarchical structure model. Standardized data can be processed by the following methods:

(7) $$\begin{array}{c} r_{ij}=\left\{\begin{array}{cc} 1, & i=1\\ \frac{1}{m}\sum_{k=1}^{m}x_{ik}\cdot x_{jk}, & i\neq j \end{array}\right\}M=\max\left(\sum_{k=1}^{m}x_{ik},x_{jk}\right). \end{array}$$

The mined information is provided to teaching decision makers to adjust teaching strategies, further guide teaching efforts and improve student performance. Use the distance formula to calculate the distance between data objects; then select the *k* data objects with the first and last connection and the largest distance product from the distance matrix as the initial clustering centroid set. The distances between other data in the data set and the *k* centroids are also calculated, and the data are classified into the classes to which the nearest data belong by comparative filtering. When the absolute value of the data is compared with the specified threshold, the soft threshold noise is removed because the part less than or equal to the threshold is zero, and the part greater than or equal to the threshold is the difference from the specified threshold. The formula is as follows:

(8) $$\begin{array}{c} \omega \lambda =\left\{\begin{array}{cc} \left[\text{sign}\left(\omega \right)\right]\left(\left|\omega \right|-\lambda \right), & \left|\lambda \right|\geq \lambda ,\\ 0, & \left|\omega \right|<\lambda , \end{array}\right.. \end{array}$$

Then, exploratory factor analysis was used to explore the dimensions of each index system level and the loadings of each factor, remove unreasonable entries, determine the formal scale, and examine the correlation coefficients between the factors of the scale. Called *X* of the whole *c* fuzzy division space. If it contains degenerate division, then it is called degenerate *c* fuzzy division space. Let

(9) $$\begin{array}{c} V=i\frac{\sum_{j=1}^{n}{\left(u_{ij}\right)}^{m}x_{j}}{\sum_{j=1}^{n}{\left(u_{ij}\right)}^{m}}. \end{array}$$

Finally, maintain the basic data needed to manage the academic affairs system, such as faculty information, major setting information, classroom information, teacher information, and textbook information. But if the result of mining is not what mining wants or the knowledge obtained is not meaningful, then it is necessary to go back to the previous stage in the process of data mining. In iterative optimization, the similarity metric can be changed and a new criterion function can be selected if necessary. Therefore, before selecting a new criterion function, the data needs to be preprocessed and transformed to be suitable for mining. Assume a certain mixed data *X*(*k*), consisting of *m* dimensional observed signal vectors.

(10) $$\begin{array}{c} \text{x}\left(k\right)={\left[x_{1}\left(k\right),x_{2}\left(k\right),\ldots ,x_{m}\left(k\right)\right]}^{T}. \end{array}$$

Based on the distance between each cluster's mean and the remaining objects, the most similar cluster is assigned. Following that, each cluster's data matrix is computed. The data matrix is often referred to as a modal matrix because its rows and columns have different meanings, whereas the dissimilarity matrix's rows and columns represent the same entities, so it is referred to as a unimodal matrix. Self-reflexivity, symmetry, and transferability must be satisfied to determine whether a fuzzy relation satisfies the equivalence relation. The relation matrix is self-reflexive when all elements of the main diagonal are 1. The symmetry of a relation matrix can be demonstrated by showing it to be a symmetric matrix. Adjust the data set after calculating the centroid values of the new symmetric matrix. We consider that we have obtained the final data clustering result if the clustering centers between the new and old classes do not change or remain within a small range.

## 4. Analysis of the Clustering Algorithm in the Higher Education Management and Student Performance Evaluation Mechanism

### 4.1. Analysis of K-Means Clustering Algorithm

**Figure 4:**
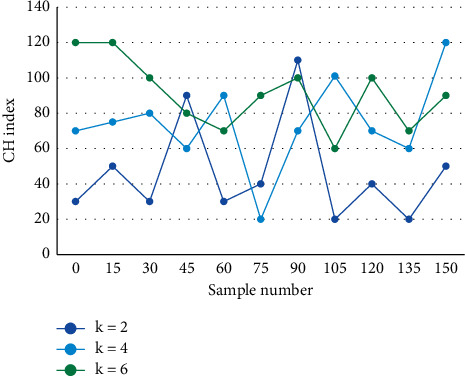
CH indicators of different *k* values.

The K-means clustering algorithm is used to deal with data clustering problems. Due to its simplicity and early introduction, the algorithm has a wide impact in scientific and industrial applications. The *K*-Means algorithm allows us to understand the learning situation of a course by analyzing the students' comprehensive examination results. According to the characteristics of this course, combined with the students' own existence, it is possible to make a reasonable and scientific evaluation of the students' learning effectiveness, providing some guidance for teaching activities, and the final results will inspire the teachers' teaching methods. After clustering students' performance using the global central clustering algorithm, the results of *k*=2,4,6 clustering were compared with CH indicators, and the relationship between the indicator values and the number of clusters is shown in Figure 4.

**Figure 5:**
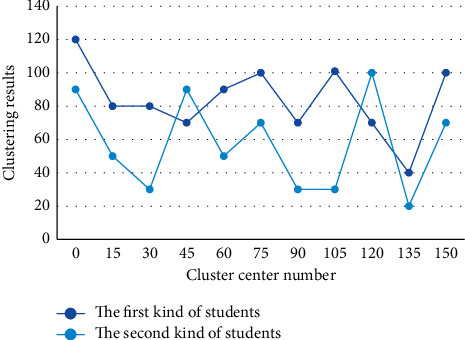
Comparison of clustering results between the first type of students and the second type of students.

Firstly, *k* objects are arbitrarily selected from n data objects as the initial clustering centers. If the data is given in the form of a data matrix, the data matrix can be converted into a phase difference matrix. A new cluster center is calculated for each assigned cluster, and then the data assignment process continues. After several iterations, if the cluster centers no longer change, it means that all data objects are assigned to their clusters and the clustering criterion function converges; otherwise, the iterative process continues until convergence. Students are divided into two categories, excellent and deviant, and the number of clusters should be close to the number of clustering variables used. In this experiment, *k*=6 was chosen. Several students' academic performance was randomly selected as the initial clustering centers through initial center of data analysis. The clustering results of the first class of students and the second class of students are shown in Figure 5.

**Figure 6:**
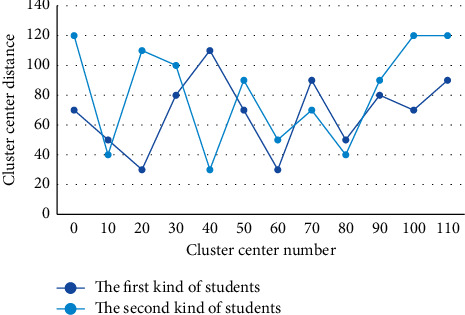
Comparison of the distance between the scores of the first class students and the second class students to each cluster center.

Select an actual object that represents the cluster. Then, based on the similarity, each remaining object is clustered into the cluster where the most similar object is located. Based on the initial clustering of the set, the sample with the smallest distance and in the class is selected as the cluster center to generate a temporary set of cluster centers. A comparison of the distances between the scores of the students in the first category and the students in the second category to each cluster center is shown in Figure 6.

Based on different classification patterns, the behavior of other data can be predicted. Obviously, the larger the value of interclass distance and criterion function, the better the separation of the various types of clustering results and therefore the higher the quality of clustering. Which distance calculation formula is used to measure the similarity between data objects needs to be chosen from the actual situation.

Next, the distance of each object to the center of each cluster is calculated and the object is assigned to the nearest cluster. Usually, clustering algorithms use the separation in characteristic space as a measurement to determine the similarity between the two samples. Suppose there is a data set containing *n* objects, and the data set is divided into k clusters by a division-based clustering method. With each sample as the center of the sphere and *d* as the radius, the number of samples that fall on the sphere is called the density of that point and is sorted by density. A batch of coalescing points is selected at first according to a certain method, and then the samples are allowed to coalesce to the nearest coalescing point so that the points coalesce into classes to obtain the initial classification. There are many data preprocessing techniques that can be used to remove noise, and for those null values, we can also use some data normalization methods that can make the clustering algorithm more accurate. Then, according to the nearest distance principle, the unreasonable classification is corrected until the classification is reasonable, thus forming the final classification result.

Finally, the centers of the *K* clusters are recalculated after all objects have been specified. The method is implemented based on the principle of minimizing the sum of the differences between the corresponding reference points of all objects. Each sample is divided into corresponding clusters according to the minimum distance, and the iterative process of clustering centers is repeated until the clustering error sum-of-squares function converges and the clustering is completed. A characteristic of this algorithm is to adjust the distribution of all data points in each iteration, and then recalculate the clustering centers into the next iteration. If the positions of all data points are unchanged in a certain iteration and the corresponding clustering centers are unchanged, it indicates that the clustering criterion function has converged and the algorithm is finished.

### 4.2. Analysis of Improved *K*-Means Algorithm

Educational data mining takes a very vital function in the process of school education, and the huge and complex educational data brings difficulties to our work. How to utilise education data better for data mining and provide better support analysis for college decision making is another major problem we face. The traditional K-means algorithm can only be used when the mean value of the class is determined; the algorithm requires the user to give *k* in advance; this algorithm is not suitable for discovering clusters with non-convex shapes or clusters with large size differences. The improved *K*-means algorithm introduces a new measure of dissimilarity to deal with the classified objects. Instead, a simple matching pattern of phase dissimilarity measures is used and corrected using a frequency-based approach during clustering to minimize the value of the clustering cost function. We collected 150 samples and conducted an experimental study of the data in these samples using the *K*-Means clustering algorithm. The number of clustering process was *k*=2 and the results after clustering analysis are shown in Table 1.

First, we find a random representative object for each cluster, and determine the *k* clusters of n data objects. At present, academic examinations for secondary school students contain many subjects, and the results are basically presented in the form of “scores”. The scores of each subject can be the same or different, usually 150 or 100 points. According to the distance axiom, four conditions of the distance axiom should be satisfied when defining the distance measure: self-similarity, minimum value, symmetry, and triangle inequality. If the distance between the attribute values calculated from the data samples is less than the set threshold, we consider these sample data as the same cluster and grouped into one category, otherwise, they are considered as different categories.

Secondly, if the quality of the obtained clusters can be improved by replacing a cluster representative, then the old cluster objects can be replaced by new ones and other objects will belong to each corresponding cluster according to their distances from these cluster representatives. Students have a well-defined interval for their exam results, with a minimum score of 0 and a maximum score of full. Each subject is measured in terms of scores within this range, and there are no cases of abnormally high or low data. The comparison of clustering time and sum of squared clustering errors using *K*-means clustering algorithm, fast global mean clustering algorithm, and the improved algorithm in this paper is shown in Table 2.

The *K*-means clustering algorithm, the fast global mean clustering algorithm, and the improved algorithm in this paper have the same clustering effect on randomly generated data sets with noisy points, but the clustering time of this algorithm is only 0.04, which has obvious advantages.

**Figure 7:**
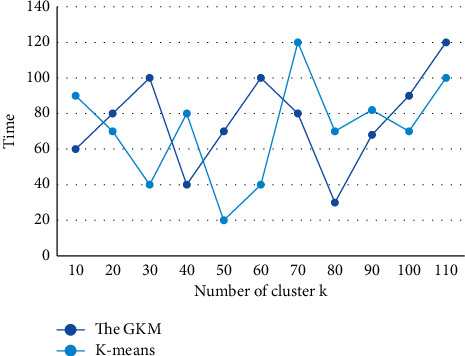
Running time of two algorithms on Iris data set with different number of categories.

**Figure 8:**
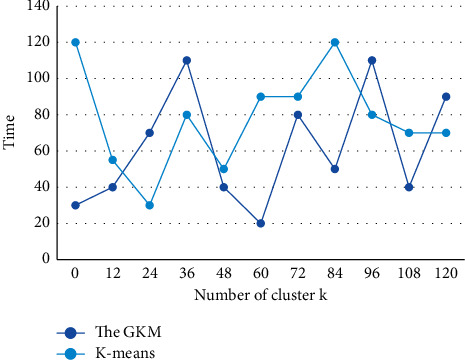
Running time of two algorithms when different categories are taken on Segmentation data set.

On the basis of the sample similarity measure, it is also necessary to determine the criterion function for evaluating the quality of the clustering results, so that the samples that really belong to the same class are aggregated into a type of subset and the samples of different classes are separated. The objects in the same cluster should be as close as possible, and the objects in different clusters should be “far away”. After clustering, the similarity between data in the same cluster, i.e., the same class of data, is extremely high, while the similarity between data in different clusters is very low, i.e., the data in the clusters are very different. In order to show the superiority of *K*-means algorithm in time, the graphs of the GKM and *K*-means algorithm running time with increasing number of clusters on two sets of data are given in Figure 7 and 8.

Finally, the clustering quality is assessed using a cost function based on the distance between each object and its clustering representative, with the change in distance variance accumulated by replacing unsuitable representatives, which is the clustering criterion function's output. The mean of the data becomes a constant 0, the standard deviation is a constant 1, and the coefficient of variation does not exist after the translation standard deviation transformation, indicating that the data resolution is completely assimilated. The clustering criterion function can also be used to assess the clustering results' quality. The clustering process should be repeated if the clustering quality does not meet the requirements. Instead of considering the entire data set, a representative sample of the data is chosen, and then the centroids are chosen from the sample.

## 5. Conclusions

The rapid development of the Internet has provided excellent technical support for the growth of online education, allowing it to overcome the time and space constraints of the existing school education system and the teacher-led education model. Clustering is an unsupervised learning algorithm that can cluster data sets without using predefined classification rules, allowing it to mine hidden information in data and create relevant data models. In the trend of education informatization, the application of data mining technology to data analysis in the teaching field will undoubtedly have a very broad perspective. Academic performance evaluation is an important component of the comprehensive evaluation reform of education quality. The use of a clustering algorithm in student performance analysis compensates for the lack of traditional evaluation methods by comparing student performance differences horizontally. Clustering analysis has evolved into a tool that can be used not only for data processing but also for data analysis when combined with other algorithms. As a result, this paper proposes a mechanistic analysis of higher education management and student performance evaluation based on clustering algorithm to assess the quality of college classroom teaching from two perspectives: students' learning effects and teachers' teaching work, with the *K*-Means algorithm as the primary method. The theory and application of clustering are highlighted based on a summary of data mining theory. This research presents a set of scientific and reasonable management capability evaluation index systems for universities, which serves as a strong foundation for relevant departments to conduct university administration capability evaluations in the future and, as a result, contributes significantly to raising the standard of university administration.

## Figures and Tables

**Table 1:** Results of clustering.

	Good	Medium	Poor
Teaching ability	27.36	19.56	9.49
Teaching method	45.22	39.54	18.40
Content of courses	44.28	17.67	10.65
Teaching attitude	39.52	29.55	18.76
Number of samples	43	57	50
Percentage	29%	38%	33%

**Table 2:** Comparison of clustering results of randomly generated data.

	*E* (×10^3^)	*T* (s)
*K*-means clustering algorithm	0.567	1.23
Fast global mean clustering algorithm	0.674	0.12
Improved *K*-means clustering algorithm	0.749	0.04

## Data Availability

The data used to support the findings of this study are available from the author upon request.
